# Understanding Illicit Prescription Painkiller Use: A Multivariable Exploration of Mental Health and Other Predictors Among Canadian University Students: Comprendre la consommation illicite d’analgésiques sur ordonnance : une analyse multivariée de la santé mentale et d’autres facteurs prédictifs chez les étudiants universitaires canadiens

**DOI:** 10.1177/07067437261462695

**Published:** 2026-06-25

**Authors:** Sanewal Singh, Viktor Prifti, Samantha Meyer

**Affiliations:** 1 School of Public Health Sciences, 8430 University of Waterloo, Waterloo, Canada; 2 Trauma Research, 10071 St Michael's Hospital, Toronto, Canada

**Keywords:** Analgésiques, étudiants universitaires, usage non médical, Canada, mésusage de médicaments, painkiller, university students, college students, nonmedical use, Canada, drug misuse

## Abstract

**Background and Objectives:**

Illicit prescription painkiller use among university students is a growing concern, influenced by a range of demographic, mental and physical health, academic, and co-substance use factors contributing to the elevated risk. However, Canadian evidence specific to university students remains limited. This study examined correlates of self-reported illicit prescription painkiller use among Canadian university students.

**Method:**

A secondary analysis of data from the American College Health Association National College Health Assessment II Canadian university edition was conducted. The dependent variable was past-year illicit prescription painkiller use, defined as a yes or no response to self-reported use of prescription painkillers without a prescription from a healthcare provider in the past 12 months. Descriptive statistics and binary logistic regression were used to examine associated demographic, academic, physical health, mental health, and co-substance use factors.

**Results:**

Among the analytical sample of 44,508, 5.8% (*n* = 2,585) reported illicit prescription painkiller use in the past 12 months. Significant predictors included race, sex, sexual identity, international student status, mental health factors, chronic illness, academic factors, and co-substance use. Higher odds of use were observed among West Asian and Black students, international students, students with chronic illness, students reporting hopelessness, feeling overwhelmed, depression with functional impairment, or suicidal ideation, and students reporting tobacco, alcohol, e-cigarette, or cocaine use.

**Conclusions:**

Illicit prescription painkiller use among Canadian university students was associated with demographic, mental health, physical health, academic, and co-substance use factors. Findings support campus-based prevention and support strategies that integrate substance use education, mental health services, and equitable access to care.

## Plain Language Summary Title

Understanding Prescription Painkiller Use Without a Prescription Among Canadian University Students: The Role of Mental Health, Student Characteristics, Physical Health, Academics, and Substance Use

Plain Language Summary

Prescription painkiller use such as opioids is an important concern among university students. This study used Canadian data from the American College Health Association National College Health Assessment II to examine which student characteristics were associated with this behaviour.

Among the analytic sample, 5.8% reported using prescription painkillers without a prescription in the past 12 months. Higher odds of use were seen among students with mental health concerns, chronic illness, international student status, and use of other substances such as tobacco, alcohol, e-cigarettes, and cocaine. Differences were also observed by race, sex, sexual identity, academic year, and grade point average.

These findings suggest that prescription painkiller use without a prescription is linked to broader health, social, and academic factors. Universities may benefit from prevention strategies that combine substance use education, mental health support, and improved access to care.

## Résumé

**Contexte et objectifs:**

La consommation illicite d’analgésiques sur ordonnance chez les étudiants universitaires est un sujet de préoccupation croissante. Elle est influencée par un éventail de facteurs démographiques, liés à la santé mentale et physique, scolaires et liés à la consommation concomitante d’autres substances psychoactives qui contribuent à l’accroissement du risque. Cependant, les données canadiennes propres aux étudiants universitaires demeurent limitées. Cette étude a examiné les facteurs associés à la consommation illicite autodéclarée d’analgésiques sur ordonnance chez les étudiants universitaires canadiens.

**Méthodes:**

Nous avons réalisé une analyse secondaire des données tirées de l’édition canadienne de l’enquête de l’*American College Health Association National College Health Assessment II*. La variable dépendante était la consommation illicite d’analgésiques sur ordonnance au cours de la dernière année, définie comme une réponse affirmative ou négative à la question portant sur la consommation autodéclarée d’analgésiques sur ordonnance sans détenir une ordonnance d’un professionnel de la santé au cours des 12 derniers mois. Les facteurs démographiques, scolaires, liés à la santé physique et mentale et liés à la consommation concomitante d’autres substances psychoactives ont été évalués à l’aide de statistiques descriptives et d’une régression logistique binaire.

**Résultats:**

Dans l’échantillon d’analyse de 44 508 personnes, 5,8 % (n = 2 585) d’entre elles ont déclaré avoir consommé de manière illicite des analgésiques sur ordonnance au cours des 12 derniers mois. Les facteurs prédictifs importants comprenaient l’origine ethnique, le sexe, l’identité sexuelle, le statut d’étudiant étranger, les facteurs liés à la santé mentale, la maladie chronique, les facteurs scolaires et la consommation concomitante de substances psychoactives. Un risque plus élevé de consommation a été observé chez les étudiants originaires de l’Asie de l’Ouest et de race noire, les étudiants étrangers, les étudiants atteints d’une maladie chronique, les étudiants ayant déclaré éprouver un sentiment de désespoir ou de surmenage, ceux atteints de dépression s’accompagnant d’altération fonctionnelle ou d’idées suicidaires, et les étudiants qui consomment du tabac, de l’alcool, des cigarettes électroniques ou de la cocaïne.

**Conclusions:**

La consommation illicite d’analgésiques sur ordonnance chez les étudiants canadiens a été associée à des facteurs démographiques, liés à la santé mentale et physique, scolaires et liés à la consommation concomitante d’autres substances psychoactives. Les résultats plaident en faveur des stratégies de prévention et de soutien mises en œuvre sur les campus, qui associent l’éducation sur la consommation de substances psychoactives, les services de santé mentale et l’accès équitable aux soins.

## Introduction

Irrespective of the many emerging global policies to limit the current prescription painkiller crisis,^1,2^ the frequency of prescriptions, overdoses, and related deaths have increased over the past decade.^3,4^ Much of this rise is believed to date back to the 1990s as a result of the billion-dollar pharmaceutical industry campaigns.^1^ In 2013, it was estimated that 207 million opioid prescriptions were written globally, an increase from the 76 million prescribed in 1991.^5^ In 2015, 4.7% of those with opioid prescriptions misused them,^6^ and treatment admissions for substance use disorders more than doubled between 2002 and 2010.^5^ Notably, approximately a third of admissions were among individuals aged 18 to 34.^5^ Young adults between the ages of 18 and 25 are particularly vulnerable to opioid use disorders, with this age group reporting the highest use in recent national surveys.^7^ Further, the risk of developing opioid use disorders tends to occur commonly among adolescents and young adults, which coincides with most college/university students.^8^ While limited research has examined painkiller misuse, a study using the fall 2008 National College Health Assessment (NCHA) data, though outdated, found that 13% of students had used prescription drugs without a prescription.^9^

Substance use during university has been associated with a range of adverse outcomes, including decreased academic performance, higher unemployment rates post-graduation, increased risk of committing or experiencing sexual assault, greater risk of engagement in illegal behaviours, psychological distress, and higher dropout rates.^5,8,10^ Mental health is a particularly relevant factor, as students with anxiety, depression, or other mental health disorders are at a greater risk of substance misuse^11^; conversely, substance use itself may worsen or trigger symptoms of anxiety and depression, suggesting a bidirectional relationship.^12,13^ To add, this age group has been found to be more reluctant to enter and retain treatment, despite experiencing significant distress.^8^ A systematic review by Weyandt et al.^10^ found that misuse of prescription opioids among students varied according to motives, co-occurring substance use, gender, and ethnicity. For instance, 31.9% of students misused opioids to get high, while 26.8% did so out of curiosity.^10^ Males and White students reported higher rates of misuse and were more likely to combine opioids with other substances such as alcohol, marijuana, and cocaine.^10^ In contrast, other studies suggest that Black individuals may be more likely to obtain and use opioids illicitly, often as a consequence of systematic barriers to healthcare services and treatment, compared to White populations.^1^ The role of international student status is mixed, while some students report lower rates of use due to cultural norms and stricter legal consequences,^14^ others suggest that increased misuse due to reduced supervision and cultural adjustments.^15^

Despite growing concerns, Canada continues to face a significant opioid crisis, with over 40,000 apparent opioid toxicity deaths recorded between 2016 and 2023.^16^ Canadian evidence also shows a substantial mortality burden among young adults aged 20 to 39 years.^17^ While this mortality data does not directly measure prescription painkiller misuse among students, they highlight the importance of studying opioid- and painkiller-related risk in young adult populations. Yet, Canadian data on painkiller misuse among students remain limited. Existing studies often include small samples,^5,18–20^ focus narrowly on either descriptive variables or specific psychological or physical factors.^18,20,21^ Furthermore, most of the existing literature on student opioid misuse is based on American university and college students, which may not directly translate to the Canadian context due to significant differences in the healthcare systems, drug policies, demographic patterns,^22–24^ and campus culture.^25^ The need for Canadian-specific data is further emphasized by Canada's high level of prescription opioid consumption.^26^

Herein, we extend existing literature, identifying the correlates of students’ illicit prescription painkiller use through multivariable analysis. We examined the specific predictors associated with the illicit use of prescription painkillers, including demographic factors, health factors, academics, and alcohol/drug use. Investigating painkiller misuse by incorporating a broader set of risk factors, particularly mental health, offers a more comprehensive assessment of vulnerabilities among university students in Canada. Given the increasing burden of mental health challenges in Canada,^27^ understanding prescription painkiller misuse risks among students has become increasingly important. Approximately 5 million Canadians met the diagnostic criteria for mood and anxiety disorders in 2022, including depression, bipolar disorder, generalized anxiety disorder, and social phobias.^28^ Our study provides a more recent reflection of user patterns for painkiller misuse within the distinct cultural, societal, and policy context of Canada. Specifically, this study aims to identify the demographic, physical health, mental health, academic, and substance use predictors of illicit prescription painkiller use among Canadian university students using a large, national dataset.

## Materials and Methods

### Survey Design and Participants

This study is a secondary analysis of data obtained from a cross-sectional, self-report survey collected by the American College Health Association (ACHA). The survey collects self-reported information on students’ general health, disease and injury prevention, academic impacts, tobacco/alcohol/drug use, sexual behaviour, and mental health. In the ACHA-NCHA II Spring 2019 Canadian Survey, the dependent variable was assessed using an item asking whether students had used “prescription painkillers,” such as OxyContin, Vicodin, Codeine, without a prescription from a healthcare provider in the past 12 months. These examples are opioid-type prescription analgesics; however, the survey did not ask respondents to identify the specific medication used. Therefore, this study uses the term “illicit prescription painkiller use” to remain consistent with the survey wording, while acknowledging that the item primarily reflects non-prescribed use of opioid-type prescription painkillers and cannot be interpreted as confirmed opioid-specific use. Research ethics approval was granted by the University of Waterloo Research Ethics Board in accordance with the Tri-Council Policy Statement for the Ethical Conduct for Research Involving Humans. All participants in this study provided voluntary, and informed consent to data collection. The study protocol adheres to the ethical principles outlined in the Declaration of Helsinki. Participant data were treated with strict confidentiality and anonymized to protect privacy. The research poses minimal risk to participants, and all procedures were approved by the institutional ethics committee.

### Variables of Interest

#### Outcome

The primary outcome was past-year illicit prescription painkiller use, defined as self-reported use of prescription painkillers without a prescription from a healthcare provider in the previous 12 months. Responses were categorized as yes or no.

#### Predictors of interest

Predictors of interest included race, international student status, academic performance, general health, chronic illness, mental health indicators, and alcohol, tobacco, e-cigarette, marijuana, and cocaine use.

#### Covariates

The multivariable model adjusted for demographic, academic, physical health, mental health, and co-substance use variables selected based on prior literature and theoretical relevance. Categories with sparse counts were collapsed where appropriate to improve model stability and interpretability.

### Data Analysis

All statistical analyses were conducted in RStudio.^29,30^ To reflect the university student population, the analytic regression sample was restricted to participants aged 18 to 30. This age range was selected to include traditional undergraduate students as well as older undergraduate, graduate, and professional students who remain relevant to university-based prevention and intervention efforts. Descriptive statistics for the overall sample and for students reporting past-year illicit prescription painkiller use were calculated and are presented in Table 1. Percentages were calculated using the available responses for each variable unless otherwise specified; therefore, percentages may not sum to 100% because of missing, skipped, or invalid responses. Difference between students who did and did not report past-year illicit prescription use were assessed using Pearson's chi-squared tests for categorical variables and Wilcoxon rank-sum tests for continuous variables, with corresponding *P*-values reported in Table 1. A multivariable binary logistic regression analysis was used to examine the association between risk factors of interest and illicit prescription painkiller use.

**Table 1.** Characteristics of Canadian University Students by Illicit Prescription Painkiller Use in the Last 12 Months (*N* = 44,508).

Characteristic	Total (*N* = 44,508)	No Illicit Painkiller Use (*N* = 41,923)	Illicit Painkiller Use (*N* = 2,585)	*P*-Value
General health				<.001
Fair	8,370 (18.8%)	7,687 (18.3%)	683 (26.4%)	
Good to excellent	34,508 (77.5%)	32,777 (78.2%)	1,731 (67.0%)	
Poor	1,630 (3.7%)	1,459 (3.5%)	171 (6.6%)	
Age (years)	21.6 (3.0)	21.6 (3.0)	21.5 (2.9)	.034
Sex				<.001
Female	31,104 (69.9%)	29,187 (69.6%)	1,917 (74.2%)	
Male	13,404 (30.1%)	12,736 (30.4%)	668 (25.8%)	
Race/ethnicity				
White	24,469 (55.0%)	23,210 (55.4%)	1,259 (48.7%)	
Aboriginal	890 (2.0%)	828 (2.0%)	62 (2.4%)	
Arab	757 (1.7%)	693 (1.7%)	64 (2.5%)	
Black	1,466 (3.3%)	1,360 (3.2%)	106 (4.1%)	
Chinese	3,189 (7.2%)	3,024 (7.2%)	165 (6.4%)	
Filipino	1,159 (2.6%)	1,112 (2.7%)	47 (1.8%)	
Japanese	53 (0.1%)	51 (0.1%)	2 (0.1%)	
Korean	430 (1.0%)	414 (1.0%)	16 (0.6%)	
Latin American	938 (2.1%)	864 (2.1%)	74 (2.9%)	
South Asian	4,811 (10.8%)	4,443 (10.6%)	368 (14.2%)	
Southeast Asian	795 (1.8%)	758 (1.8%)	37 (1.4%)	
West Asian	435 (1.0%)	379 (0.9%)	56 (2.2%)	
Multiracial	4,171 (9.4%)	3,916 (9.3%)	255 (9.9%)	
Other	945 (2.1%)	871 (2.1%)	74 (2.9%)	
International student				<.001
No	38,555 (86.6%)	36,464 (87.0%)	2,091 (80.9%)	
Yes	5,953 (13.4%)	5,459 (13.0%)	494 (19.1%)	
Marital status				.2
Single	38,702 (87.0%)	36,489 (87.0%)	2,213 (85.6%)	
Married/partnered	4,165 (9.4%)	3,900 (9.3%)	265 (10.3%)	
Divorced/separated	190 (0.4%)	175 (0.4%)	15 (0.6%)	
Other	1,451 (3.3%)	1,359 (3.2%)	92 (3.6%)	
Residence				
Parent/guardian's home	15,788 (35.5%)	14,845 (35.4%)	943 (36.5%)	
Campus residence hall	5,410 (12.2%)	5,108 (12.2%)	302 (11.7%)	
Fraternity or sorority house	74 (0.2%)	63 (0.2%)	11 (0.4%)	
Other college/university housing	935 (2.1%)	862 (2.1%)	73 (2.8%)	
Other off-campus housing	22,301 (50.1%)	21,045 (50.2%)	1,256 (48.6%)	
Year in school				<.001
Graduate or professional	4,693 (10.5%)	4,460 (10.6%)	233 (9.0%)	
1st year undergraduate	11,685 (26.3%)	10,888 (26.0%)	797 (30.8%)	
2nd year undergraduate	10,522 (23.6%)	9,878 (23.6%)	644 (24.9%)	
3rd year undergraduate	7,698 (17.3%)	7,318 (17.5%)	380 (14.7%)	
4th year undergraduate	5,724 (12.9%)	5,419 (12.9%)	305 (11.8%)	
5th year or more undergraduate	2,339 (5.3%)	2,217 (5.3%)	122 (4.7%)	
Other	1,847 (4.1%)	1,743 (4.2%)	104 (4.0%)	
GPA				<.001
B	20,081 (45.1%)	18,865 (45.0%)	1,216 (47.0%)	
A	17,013 (38.2%)	16,243 (38.7%)	770 (29.8%)	
C	6,786 (15.2%)	6,250 (14.9%)	536 (20.7%)	
D/F	628 (1.4%)	565 (1.3%)	63 (2.4%)	
Weight perception				<.001
About the right weight	23,452 (52.7%)	22,278 (53.1%)	1,174 (45.4%)	
Very underweight	465 (1.0%)	419 (1.0%)	46 (1.8%)	
Slightly underweight	4,737 (10.6%)	4,440 (10.6%)	297 (11.5%)	
Slightly overweight	13,824 (31.1%)	12,914 (30.8%)	910 (35.2%)	
Very overweight	2,030 (4.6%)	1,872 (4.5%)	158 (6.1%)	
Chronic illness				<0.001
No	42,154 (94.7%)	39,770 (94.9%)	2,384 (92.2%)	
Yes	2,354 (5.3%)	2,153 (5.1%)	201 (7.8%)	
Sexual orientation				<.001
Straight/heterosexual	36,786 (82.7%)	34,844 (83.1%)	1,942 (75.1%)	
Bisexual	3,887 (8.7%)	3,520 (8.4%)	367 (14.2%)	
Gay	627 (1.4%)	602 (1.4%)	25 (1.0%)	
Lesbian	494 (1.1%)	453 (1.1%)	41 (1.6%)	
Other	2,714 (6.1%)	2,504 (6.0%)	210 (8.1%)	
Felt hopeless				<.001
No, never	8,453 (19.0%)	8,227 (19.6%)	226 (8.7%)	
No, not in the last 12 months	6,924 (15.6%)	6,652 (15.9%)	272 (10.5%)	
Yes, in the last 12 months	29,131 (65.5%)	27,044 (64.5%)	2,087 (80.7%)	
Felt overwhelmed				<.001
No, never	2,862 (6.4%)	2,780 (6.6%)	82 (3.2%)	
No, not in the last 12 months	1,687 (3.8%)	1,608 (3.8%)	79 (3.1%)	
Yes, in the last 12 months	39,959 (89.8%)	37,535 (89.5%)	2,424 (93.8%)	
Depressed—difficulty functioning				<.001
No, never	12,902 (29.0%)	12,526 (29.9%)	376 (14.5%)	
No, not in the last 12 months	8,227 (18.5%)	7,849 (18.7%)	378 (14.6%)	
Yes, in the last 12 months	23,379 (52.5%)	21,548 (51.4%)	1,831 (70.8%)	
Overwhelming anxiety				<.001
No, never	8,359 (18.8%)	8,104 (19.3%)	255 (9.9%)	
No, not in the last 12 months	4,804 (10.8%)	4,569 (10.9%)	235 (9.1%)	
Yes, in the last 12 months	31,345 (70.4%)	29,250 (69.8%)	2,095 (81.0%)	
Suicide ideation				<.001
No, never	29,054 (65.3%)	27,824 (66.4%)	1,230 (47.6%)	
No, not in the last 12 months	7,816 (17.6%)	7,286 (17.4%)	530 (20.5%)	
Yes, in the last 12 months	7,638 (17.2%)	6,813 (16.3%)	825 (31.9%)	
Difficulty: academics				<.001
No	17,311 (38.9%)	16,562 (39.5%)	749 (29.0%)	
Yes	27,197 (61.1%)	25,361 (60.5%)	1,836 (71.0%)	
Difficulty: intimate relationships				<.001
No	29,197 (65.6%)	27,843 (66.4%)	1,354 (52.4%)	
Yes	15,311 (34.4%)	14,080 (33.6%)	1,231 (47.6%)	
Difficulty: family problems				<.001
No	28,536 (64.1%)	27,255 (65.0%)	1,281 (49.6%)	
Yes	15,972 (35.9%)	14,668 (35.0%)	1,304 (50.4%)	
Difficulty: personal health				<.001
No	30,895 (69.4%)	29,537 (70.5%)	1,358 (52.5%)	
Yes	13,613 (30.6%)	12,386 (29.5%)	1,227 (47.5%)	
Stress level				<.001
Average stress	13,301 (29.9%)	12,738 (30.4%)	563 (21.8%)	
No stress	792 (1.8%)	750 (1.8%)	42 (1.6%)	
Less than average stress	3,054 (6.9%)	2,922 (7.0%)	132 (5.1%)	
More than average stress	20,735 (46.6%)	19,488 (46.5%)	1,247 (48.2%)	
Tremendous stress	6,626 (14.9%)	6,025 (14.4%)	601 (23.2%)	
Cigarette use, last 30 days				<.001
Never used	32,917 (74.3%)	31,402 (75.2%)	1,515 (58.9%)	
Used 1-5 days	2,215 (5.0%)	1,981 (4.7%)	234 (9.1%)	
Used 20+ days	1,530 (3.5%)	1,330 (3.2%)	200 (7.8%)	
Used 6-19 days	820 (1.8%)	695 (1.7%)	125 (4.9%)	
Used, but not in last 30 days	6,848 (15.4%)	6,351 (15.2%)	497 (19.3%)	
Alcohol use, last 30 days				<.001
Never used	8,178 (18.4%)	7,782 (18.6%)	396 (15.3%)	
Used 1-5 days	17,514 (39.4%)	16,593 (39.6%)	921 (35.7%)	
Used 20+ days	1,525 (3.4%)	1,351 (3.2%)	174 (6.7%)	
Used 6-19 days	9,863 (22.2%)	9,186 (21.9%)	677 (26.2%)	
Used, but not in last 30 days	7,355 (16.6%)	6,942 (16.6%)	413 (16.0%)	
Marijuana use, last 30 days				<.001
Never used	23,813 (53.6%)	22,649 (54.1%)	1,164 (45.1%)	
Used 1-5 days	6,261 (14.1%)	5,883 (14.1%)	378 (14.7%)	
Used 20+ days	2,663 (6.0%)	2,357 (5.6%)	306 (11.9%)	
Used 6-19 days	2,695 (6.1%)	2,442 (5.8%)	253 (9.8%)	
Used, but not in last 30 days	8,984 (20.2%)	8,505 (20.3%)	479 (18.6%)	
Cocaine use, last 30 days				
Never used	41,147 (92.7%)	39,017 (93.3%)	2,130 (82.6%)	
Used 1-5 days	624 (1.4%)	514 (1.2%)	110 (4.3%)	
Used 20+ days	47 (0.1%)	31 (0.1%)	16 (0.6%)	
Used 6-19 days	145 (0.3%)	108 (0.3%)	37 (1.4%)	
Used, but not in last 30 days	2,443 (5.5%)	2,156 (5.2%)	287 (11.1%)	
Methamphetamine use, last 30 days				
Never used	43,704 (98.4%)	41,254 (98.6%)	2,450 (95.1%)	
Used 1-5 days	72 (0.2%)	56 (0.1%)	16 (0.6%)	
Used 20+ days	34 (0.1%)	21 (0.1%)	13 (0.5%)	
Used 6-19 days	40 (0.1%)	28 (0.1%)	12 (0.5%)	
Used, but not in last 30 days	552 (1.2%)	466 (1.1%)	86 (3.3%)	
E-cigarette use, last 30 days				<.001
Never used	34,226 (77.4%)	32,538 (78.1%)	1,688 (65.8%)	
Used 1-5 days	2,413 (5.5%)	2,210 (5.3%)	203 (7.9%)	
Used 20+ days	1,763 (4.0%)	1,551 (3.7%)	212 (8.3%)	
Used 6-19 days	943 (2.1%)	851 (2.0%)	92 (3.6%)	
Used, but not in last 30 days	4,889 (11.1%)	4,517 (10.8%)	372 (14.5%)	

## Results

### Descriptive Results

Of the 55,284 respondents who completed the survey, 54,895 responded to the question on illicit painkiller use. After restricting the sample to participants aged 18 to 30 with complete data on all model covariates, the final analytic sample included 44,508 students, of whom 2,585 (5.8%) reported use in the last 12 months. The analytic sample had a mean age of 21.6 years (*SD* = 3.0) and was 69.9% female (Table 1). Among students who reported use, the majority were female (74.2%), White (48.7%), and reported good to excellent health (67.0%). In this group, 30.8% were in their first year of undergraduate studies and 19.1% were international students. Nearly half reported a B average (47.0%), while 29.8% reported an A average and 2.4% reported a D or F average (Table 1).

### Binary Logistic Regression Analysis

#### Demographic factors

**Figure 1.**
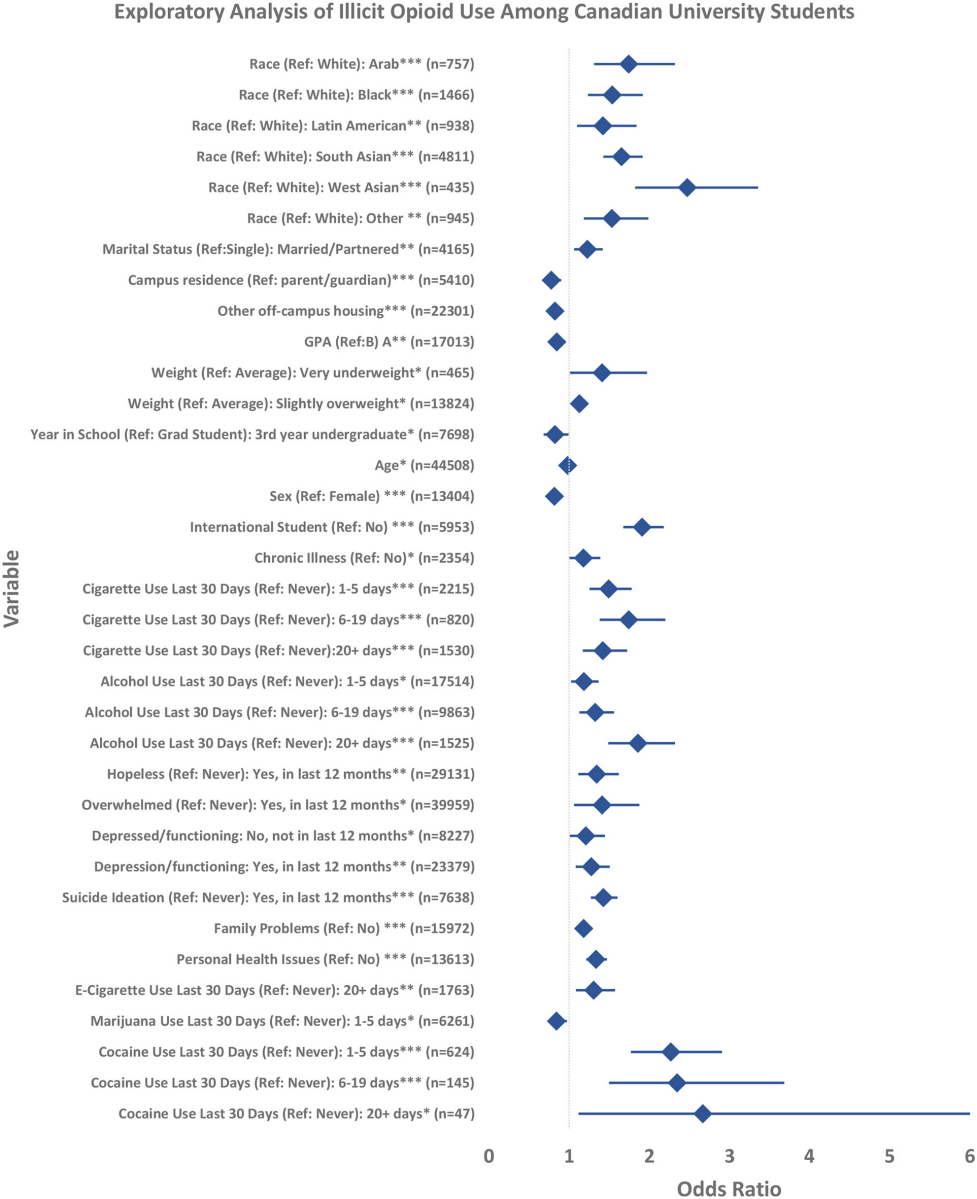
Significant predictors of illicit prescription painkiller use among Canadian university students, multivariable logistic regression (analytic sample *N* = 44,508; outcome events = 2,585). Odds ratios with 95% confidence intervals shown. Statistical significance is indicated as: *p* < .05 (*), *p* < .01 (**), and *p* < *.001* (***).

Figure 1 presents the multivariable logistic regression results, highlighting significant predictors of illicit painkiller use among Canadian university students. Findings of all covariates can be found in Appendix Table 1. Compared to White students, several racial minorities had higher odds of illicitly using painkillers, including West Asian (OR = 2.47, 95% CI, 1.82 to 3.36, *P* < .001), Arab (OR = 1.74, 95% CI, 1.31 to 2.32, *P* < .001), and Black students (OR = 1.54, 95% CI, 1.23 to 1.92, *P* < .001). Male students had lower odds of illicit prescription painkiller use than female students (OR = 0.82, 95% CI, 0.74 to 0.91, *P* < .001), as did older students (OR = 0.98, 95% CI, 0.96 to 1.00, *P* < .05). Students identifying as bisexual had higher odds of illicit use (OR = 1.29, 95% CI, 1.13 to 1.46, *P* < .001), compared to straight students.

#### Physical and mental health-related factors

The results indicate that covariates related to participants’ mental health play an important role in illicit painkiller use. Students who reported seriously considering suicide in the last 12-months had higher odds of illicit painkiller use (OR = 1.43, 95% CI, 1.27 to 1.60, *P* < .001), compared to those who reported never having such thoughts. Similarly, individuals who reported feeling hopeless (OR = 1.34, 95% CI, 1.12 to 1.62, *P* < .01), overwhelmed (OR = 1.41, 95% CI, 1.06 to 1.88, *P* < .05), or depressed to the point of impaired function (OR = 1.28, 95% CI, 1.08 to 1.51, *P* < .01) had higher odds of use. Compared with students who did not report traumatic events, those reporting trauma related to family (OR = 1.18, 95% CI, 1.08 to 1.30, *P* < .001) and personal health (OR = 1.34, 95% CI, 1.21 to 1.47, *P* < .001) also had higher odds of use. Students with chronic illnesses had higher odds of illicit painkiller use than students reporting no chronic illness (OR = 1.18, 95% CI, 1.00 to 1.39, *P* < .05).

#### Academic factors

Compared with graduate and professional students, third-year students had lower odds of nonmedical prescription painkiller use (OR = 0.82, 95% CI, 0.68 to 0.99, *P* < .05). Students with an average A GPA also had lower odds of use than students with a B average (OR = 0.85, 95% CI, 0.77 to 0.94, *P* < .01). International students had nearly twice the odds of illicit prescription painkiller use compared with domestic students (OR = 1.91, 95% CI, 1.68 to 2.18, *P* < .001).

#### Co-substance use

Co-substance use variables were strongly associated with illicit prescription painkiller use. Compared with non-smokers, students who smoked 1 to 5 days (OR = 1.49, 95% CI, 1.25 to 1.78, *P* < .001), 6 to 19 days (OR = 1.74, 95% CI, 1.38 to 2.20, *P* < .001), and more than 20 days (OR = 1.42, 95% CI, 1.17 to 1.72, *P* < .001) all had increased odds of use. Alcohol use and e-cigarettes also depicted a dose–response relationship, with individuals who drank more than 20 days (OR = 1.86, 95% CI, 1.49 to 2.32, *P* < .001) and used e-cigarettes for more than 20 days (OR = 1.31, 95% CI, 1.09 to 1.57, *P* < .001) also had increased odds, compared to non-users. Cocaine use was also associated with higher odds, with the highest estimate among students reporting cocaine use on more than 20 days (OR = 2.67, 95% CI, 1.12 to 6.37, *P* < .05). In contrast, students reporting marijuana 1 to 5 days (OR = 0.84, 95% CI, 0.73 to 0.97, *P* < .05) or previous marijuana use but not in the last 30 days (OR = 0.83, 95% CI, 0.73 to 0.95, *P* < .01) had lower odds of illicit prescription painkiller use than non-users.

## Discussion

### Race and Other Demographics

This study reveals critical insights into the prevalence and risk factors of painkiller use without a prescription among Canadian university students. Notably, the results underscore the association between race and prescription painkiller misuse. West Asian students demonstrated higher odds of painkiller misuse compared with White students. This finding should be interpreted cautiously, as the ACHA-NCHA II did not measure medication source, pain severity, cultural norms, and other important confounders. While the specific drivers of this disparity cannot be determined from cross-sectional data, the findings may reflect unmeasured structural or contextual factors, including barriers to healthcare access, differences in pain management experiences, stigma, or differential access to formal medical care. Future qualitative and longitudinal quantitative research is needed to better understand the mechanisms underlying this association.

Similarly, Black students were also more likely to use prescription painkillers without a prescription, in comparison to White students. This finding should be interpreted in the context of broader evidence on racial inequities in pain assessment, prescribing, and healthcare access. Data from 1993 to 2009 indicates large disparities between the rate of prescription painkillers, such as opiates, between White Americans (approximately 16/100,000) and Black Americans (5/100,000),^31^ highlighting disparities in access. Overall, White students were less likely to use prescription painkillers without a prescription in comparison to Black, Arab, Latin American, and West Asian students. These findings are inconsistent with existing American literature that has reported higher rates of opioid misuse among White individuals.^32^ This discrepancy may reflect important contextual differences between Canada and the United States, including disparities in healthcare access, drug policy,^33,34^ and the racial composition of the university population,^35^ which may alter who is more likely to seek illicit alternatives to prescribed medications.

Given the limited access to prescription medications often reported by minority groups, White students may have greater access to prescription painkillers through healthcare providers, making them less likely to obtain these drugs without a prescription. Moreover, doctors may be more willing to prescribe painkillers to White than to Black and minority students, as the latter groups are often incorrectly stereotyped as more likely to abuse or sell prescription painkillers.^1^ However, because the present study did not measure pain severity, prescription access, medication source, or experiences of discrimination, these mechanisms cannot be directly measured. Nonetheless, these findings highlight the importance of equity-focused prevention strategies and improved access to culturally responsive health and counselling services for racialized students.

Findings indicate that bisexual students were at a higher risk of painkiller use, consistent with previous research. This may be explained by the cognitive dissonance theory, where conflicting beliefs cause psychological distress, that may contribute to opioid misuse.^36^ Additionally, men had significantly lower odds of illicit painkiller use compared to women, aligning with studies citing differences in pain experiences, cultural norms, and likelihood of experiencing dependency.^37^ These findings suggest the need for targeted support addressing the diverse social and psychological factors influencing prescription drug misuse, particularly among bisexual students and women.

#### Physical and psychological factors

Psychological well-being was associated with illicit prescription painkiller use. Students who reported feeling hopeless, overwhelmed, having personal health issues, and those who seriously considered suicide were more likely to use prescription painkillers without a prescription compared to those who reported never having such feelings. These findings align with previous literature linking opioid misuse to suicidal ideation, depression, and psychological distress.^38–40^ However, given that our study uses cross-sectional data, we cannot determine whether mental health concerns preceded, followed, or co-occurred with illicit prescription painkiller use.

Similarly, emotional stressors such as family problems were associated with increased odds of misuse, consistent with prior research.^41^ These findings are relevant in Canadian universities given the increased prevalence of student mental health struggles and lack of university mental wellness initiatives.^42^ Canadian university students face long wait times, limited access to culturally competent care, and inadequate mental health infrastructures both on campus and within the broader healthcare system, where many even lack a family doctor.^43,44^ These disparities severely limit struggling students’ access to early preventative intervention and may contribute to their self-medication with illicitly obtained painkillers. Our findings further highlight the need for improved care to limit the risk of painkiller misuse among undergraduates.

Students with chronic illness had higher odds of illicit prescription painkiller use than those without chronic illness. Chronic pain and illness can affect academic functioning through missed class, reduced concentration, and reliance on others^45^; however, this study did not measure pain severity, prescribed medication access, or reasons for use. Students with bodyweight extremes also had higher odds of use. This association may reflect unmeasured physical discomfort, chronic health conditions, or psychological distress, but these mechanisms were not directly assessed. Additional research is needed to clarify the relationship between physical health, pain, bodyweight, and illicit prescription painkiller use among university students.

#### Academic factors

Academic performance and health-related challenges were important factors in illicit prescription painkiller use among university students. Previous studies have linked illicit drug use with lower academic performance.^5,8,10,46^ In this study, students with an A average had lower odds of use than students with a B average, while lower GPA categories did not differ significantly. This may suggest that academically high-achieving students benefit from healthier coping mechanisms, consistent with research on stress-coping profiles.^47^ Similarly, third-year students showed lower risk of misuse compared to other students. However, this finding should be interpreted cautiously as the year in school may reflect academic pressure, student responsibilities, or other unmeasured factors.

International student status was associated with higher odds of illicit prescription painkiller use. In comparison to other countries, such as America, Canadian universities have the second-highest proportion of international students, after Australia.^48^ While some research suggests that international students are less likely to misuse substances,^14,49^ a systematic review by Aresi et al.^15^ found that intercultural stressors may increase the risk of alcohol and drug use. Our findings support the latter, indicating higher odds of painkiller misuse among international students. In the Canadian context, barriers to healthcare access, communication challenges, unfamiliarity with health systems, and insurance differences may contribute to unmet health needs.^50–52^ In Canada, many international students are not covered by provincial healthcare (e.g., Ontario Health Insurance Plan),^53^ and may seek alternative forms of pain management, increasing the risk of illicit painkiller use.

#### Co-substance use

Another influential predictor of prescription painkiller uses without a prescription found in this study was a student's use of tobacco, alcohol, and other drugs. Various studies suggest that those who misuse prescription painkillers are more likely to report recreational use of other prescriptions, as well as an increased odds for engaging in cigarette smoking, heavy drinking, and other illicit drugs.^54–56^ Generally, these findings align with the results of our study that indicate increased odds of prescription painkiller use for those who had higher frequencies of drug, alcohol, and tobacco use compared to nonusers. Interestingly, while some research has shown that marijuana use is associated with higher rates of prescription painkiller misuse,^57^ other studies, including our own, have found an inverse relationship.^58^ It may be possible that students use marijuana for pain relief,^59,60^ and therefore, may not need prescription painkillers.

## Limitations and Implications of Findings

The ACHA data had a relatively low response rate (∼20%), introducing potential participation bias and limiting generalizability. Given that there are many variables of interest and there are no identifying variables (participant identifiers), no sample weights were conducted. The study utilized a narrow definition of prescription misuse as using medication without a prescription. A more inclusive definition that may include additional methods of misuse such as using another person's prescription or using one's medication for a different purpose or dose than intended. This definition of prescription misuse may limit the generalizability of our predictors. Although the ACHA-NCHA II item provided opioid-type examples of prescription painkillers, the survey did not capture the exact medication used; therefore, findings should be interpreted as illicit prescription painkiller use rather than confirmed opioid-specific misuse. Furthermore, as a cross-sectional study using data collected at a single point in time, causal relationships cannot be inferred or established. Therefore, the associations identified in this study are observational and should be interpreted accordingly. Residual confounding from unmeasured variables also cannot be ruled out, as the model may not have captured all relevant predictors of illicit painkiller use. Lastly, given that this study utilizes a self-report measure, the question requires individuals to admit to illicit drug use and may therefore be underreported, which may affect the precision of the prevalence estimates due to self-report bias.

Irrespective of these limitations, our study offers important Canada-specific insights into prescription painkiller misuse among university students, an area where national data has been limited. While Canada's publicly funded healthcare system reduces some barriers to prescription access, illicit use persists,^61^ suggesting that broader cultural and systemic factors may also contribute. Our findings underscore academic, physical, and mental health factors as key predictors of painkiller misuse, emphasizing the importance of addressing psychological and healthcare access challenges unique to the Canadian context.

Amid growing concerns of student mental health and the opioid crisis in Canada, these findings provide meaningful guidance for campus policies and public health planning. They highlight the need for culturally competent prevention, targeted outreach, and improved counselling services for high-risk populations. Targeted educational campaigns, delivered through orientation events, seminars, university newsletters, and course modules, alongside mental health initiatives, may be especially effective for incoming and at-risk students. These strategies may help students recognize the risks of illicit prescription painkiller use, identify early warning signs, and connect with national support initiatives such as the Public Health Agency of Canada's Youth Substance Use Prevention Program.

Importantly, this study does not assume the motivations behind non-medical use of prescription painkillers or suggest that all use is recreational. Instead, it identifies students who may be more vulnerable due to physical health issues, psychological distress, or limited access to care. Future research may build on this work using longitudinal designs to explore the temporal trends and risk factors over time. Ultimately, we hope that these findings support early intervention efforts and guide universities and policymakers in expanding access to timely medical and mental health support.

## Appendix

**Appendix Table 1:** Full multivariable logistic regression results predicting illicit prescription painkiller use among Canadian university students (analytic sample *N* = 44,508; outcome events = 2,585).

Variable/level	*n*	OR	95% CI	*P*-Value
Race/ethnicity (Ref: white)	24,469			
Aboriginal	890	0.98	0.74–1.30	.897
Arab***	757	1.74	1.31–2.32	<.001
Black***	1,466	1.54	1.23–1.92	<.001
Chinese	3,189	1.18	0.98–1.42	.076
Filipino	1,159	0.76	0.56–1.03	.079
Japanese	53	0.54	0.13–2.33	.412
Korean	430	0.77	0.46–1.28	.304
Latin American**	938	1.42	1.10–1.84	.007
South Asian***	4,811	1.65	1.43–1.92	<.001
Southeast Asian	795	0.84	0.60–1.19	.329
West Asian***	435	2.47	1.82–3.36	<.001
Multiracial	4,171	1.03	0.89–1.19	.715
Other**	945	1.53	1.18–1.99	.001
Marital status (Ref: single)	38,702			
Married/partnered**	4,165	1.23	1.06–1.42	.006
Divorced/separated	190	0.81	0.45–1.45	.470
Other	1,451	1.04	0.83–1.30	.724
Residence (Ref: parent/guardian's home)	15,788			
Campus residence hall***	5,410	0.78	0.67–0.90	.001
Fraternity or sorority house	74	1.00	0.44–2.26	1.000
Other college/university housing	935	1.17	0.90–1.52	.251
Other off-campus housing***	22,301	0.82	0.74–0.91	<.001
GPA (Ref: B)	20,081			
A**	17,013	0.85	0.77–0.94	.001
C	6,786	1.10	0.99–1.23	.089
D/F	628	1.00	0.75–1.33	.982
Weight perception (Ref: about the right weight)	23,452			
Very underweight*	465	1.41	1.01–1.97	.043
Slightly underweight	4,737	1.06	0.93–1.22	.388
Slightly overweight*	13,824	1.13	1.03–1.24	.012
Very overweight	2,030	1.09	0.90–1.31	.369
Year in school (Ref: graduate/professional)	4,693			
1st year undergraduate	11,685	1.16	0.96–1.39	.117
2nd year undergraduate	10,522	1.02	0.85–1.22	.855
3rd year undergraduate*	7,698	0.82	0.68–0.99	.043
4th year undergraduate	5,724	0.96	0.79–1.16	.641
5th year or more undergraduate	2,339	0.85	0.67–1.08	.185
Other	1,847	0.90	0.70–1.16	.424
Moderate exercise, last 7 days (Ref: 0 days)	11,647			
1–3 days	21,379	1.05	0.95–1.16	.324
4 or more days	11,482	1.08	0.96–1.21	.219
Age (years), per 1-year increase*	44,508	0.98	0.96–1.00	.047
Sex (Ref: female)	31,104			
Male***	13,404	0.82	0.74–0.91	<.001
International student (Ref: no)	38,555			
Yes***	5,953	1.91	1.68–2.18	<.001
Chronic illness (Ref: no)	42,154			
Yes*	2,354	1.18	1.00–1.39	.048
General health (Ref: fair)	8,370			
Good to excellent	34,508	0.93	0.84–1.03	.144
Poor	1,630	1.00	0.83–1.21	.990
Sexual orientation (Ref: straight/heterosexual)	36,786			
Bisexual***	3,887	1.29	1.13–1.46	<.001
Gay	627	0.76	0.50–1.14	.185
Lesbian	494	1.29	0.92–1.81	.139
Other	2,714	1.13	0.96–1.32	.139
Stress level (Ref: average stress)	13,301			
No stress	792	1.23	0.83–1.82	.296
Less than average stress	3,054	1.14	0.93–1.41	.204
More than average stress	20,735	1.03	0.92–1.16	.588
Tremendous stress	6,626	1.10	0.95–1.27	.214
Felt hopeless (Ref: no, never)	8,453			
No, not in last 12 months	6,924	1.07	0.87–1.33	.506
Yes, in last 12 months**	29,131	1.34	1.12–1.62	.002
Felt overwhelmed (Ref: No, never)	2,862			
No, not in last 12 months	1,687	1.33	0.93–1.91	.118
Yes, in last 12 months*	39,959	1.41	1.06–1.88	.018
Depressed—difficulty functioning (Ref: no, never)	12,902			
No, not in last 12 months*	8,227	1.21	1.01–1.45	.039
Yes, in last 12 months**	23,379	1.28	1.08–1.51	.004
Overwhelming anxiety (Ref: no, never)	8,359			
No, not in last 12 months	4,804	1.13	0.90–1.40	.291
Yes, in last 12 months	31,345	0.99	0.83–1.20	.950
Suicide ideation (Ref: no, never)	29,054			
No, not in last 12 months	7,816	1.12	1.00–1.26	.061
Yes, in last 12 months***	7,638	1.43	1.27–1.60	<.001
Difficulty: academics (Ref: no)	17,311			
Yes	27,197	0.97	0.87–1.08	.594
Difficulty: intimate relationships (Ref: no)	29,197			
Yes**	15,311	1.16	1.06–1.27	.002
Difficulty: family problems (Ref: no)	28,536			
Yes***	15,972	1.18	1.08–1.30	<.001
Difficulty: personal health issues (Ref: no)	30,895			
Yes***	13,613	1.34	1.21–1.47	<.001
Cigarette use, last 30 days (Ref: never used)	32,917			
Used, but not in last 30 days***	6,848	1.25	1.10–1.43	.001
1–5 days***	2,215	1.49	1.25–1.78	<.001
6–19 days***	820	1.74	1.38–2.20	<.001
20+ days***	1,530	1.42	1.17–1.72	<.001
Alcohol use, last 30 days (Ref: never used)	8,178			
Used, but not in last 30 days**	7,355	1.23	1.05–1.44	.009
1–5 days*	17,514	1.19	1.03–1.37	.021
6–19 days***	9,863	1.33	1.13–1.56	.001
20+ days***	1,525	1.86	1.49–2.32	<.001
E-Cigarette Use, last 30 days (Ref: Never used)	34,226			
Used, but not in last 30 days*	4,889	1.15	1.00–1.33	.047
1–5 days	2,413	1.18	0.99–1.40	.073
6–19 days	943	1.10	0.85–1.42	.458
20 + days**	1,763	1.31	1.09–1.57	.005
Marijuana use, last 30 days (Ref: never used)	23,813			
Used, but not in last 30 days**	8,984	0.83	0.73–0.95	.005
1–5 days*	6,261	0.84	0.73–0.97	.019
6–19 days	2,695	1.10	0.93–1.31	.273
20+ days	2,663	1.10	0.93–1.31	.267
Cocaine use, last 30 days (Ref: never used)	41,147			
Used, but not in last 30 days***	2,443	1.68	1.43–1.97	<.001
1–5 days***	624	2.27	1.77–2.91	<.001
6–19 days***	145	2.35	1.50–3.68	<.001
20+ days*	47	2.67	1.12–6.37	.027
Methamphetamine use, last 30 days (Ref: never used)	43,704			
Used, but not in last 30 days*	552	1.34	1.02–1.75	.034
1–5 days	72	1.38	0.72–2.64	.328
6–19 days	40	1.76	0.79–3.92	.170
20+ days	34	1.81	0.65–5.00	.255

*Significance: ***P* *<* *.001, **P* *<* *.01, *P* *<* *.05.*
